# Recent Advances on Sensor Technologies for the Monitoring of Tumor Markers

**DOI:** 10.3390/molecules31111919

**Published:** 2026-06-02

**Authors:** Yubang Dong, Qi Zhao, Yining Feng, Weikang Yang, Bo Wang, Yuqing Wang, Mingyuan Gao, Jie Zhang, Tianzhu Guan

**Affiliations:** 1 College of Medicine and Biological Information Engineering, Northeastern University, Shenyang 110016, China; 2 School of Food Science and Engineering, Yangzhou University, Yangzhou 225127, China; 3 College of Food Science and Engineering, Jilin University, Changchun 130062, China

**Keywords:** sensor technologies, tumor markers, detection and monitoring, future prospects

## Abstract

Sensor technologies have been increasingly recognized as a cornerstone for advancing tumor diagnostics amid the global health challenge posed by cancer. Traditional diagnostic methods are often constrained by inherent tumor heterogeneity, while liquid biopsy has emerged as a transformative minimally invasive alternative, with biosensors playing a pivotal role in its clinical translation. This review summarizes the progress of tumor diagnostic biosensors, focusing on electrochemical and fluorescent sensors. Electrochemical sensors excel in quantitative precision, miniaturization, and point-of-care (POCT) applicability, enabling ultra-sensitive detection of biomarkers such as circulating tumor cells, circulating tumor DNA, and exosomes through nanomaterial modification and signal amplification strategies. Fluorescent sensors, meanwhile, offer superior multiplexing capability and in situ imaging performance, which are further enhanced by novel nanomaterials. Additionally, it covers other promising sensor types including Surface-Enhanced Raman Scattering, microfluidic, photoelectrochemical, field-effect transistor, and clustered regularly interspaced short palindromic repeats and CRISPR-associated proteins-based sensors. Current research efforts are concentrated on multiplexed detection, point-of-care integration, and translation toward higher-order clinical functions such as cancer subtype discrimination, risk stratification, and prognosis. Future directions will focus on multimodal integration, intelligent data analysis, and prospective clinical validation against hard endpoints to facilitate the implementation of precision oncology.

## 1. Introduction

Cancer remains a formidable global health challenge, constituting a leading cause of morbidity and mortality. The year 2020 witnessed approximately 19.3 million new cases and 10 million deaths worldwide, underscoring the urgent need for transformative diagnostic paradigms [1]. A fundamental obstacle lies in the inherent temporal and spatial heterogeneity of malignancies, where tumor composition and molecular profiles evolve over time and vary across locations [2]. Traditional diagnostic pillars include anatomic imaging (computed tomography, magnetic resonance imaging, positron emission tomography, and ultrasound), invasive tissue biopsy, and conventional bulk assays such as enzyme-linked immunosorbent assay (ELISA) and polymerase chain reaction (PCR). While invaluable, these methods are often constrained in their ability to capture dynamic molecular complexity. Anatomic imaging resolves macroscopic lesions but provides limited molecular information; tissue biopsy yields a single localized snapshot. We note that this critique applies to macroscopic anatomic imaging and is conceptually distinct from the molecular fluorescence imaging enabled by fluorescent biosensors discussed later in this review, which operates at sub-cellular resolution and reports dynamic biochemical activity (e.g., telomerase, miRNA, microenvironmental redox state) in real time [3].

Tumor markers are biomolecules whose presence or altered abundance in body fluids reflects the existence, type, or progression of malignancy, and they serve as the central analytes of clinical oncology surveillance. They span several molecular classes. Glycoprotein antigens such as carcinoembryonic antigen (CEA), alpha-fetoprotein (AFP), prostate-specific antigen (PSA), CA125, CA15-3, CA19-9, and human epidermal growth factor receptor 2 (HER2) remain the cornerstone of routine serum-based screening and recurrence monitoring. Nucleic-acid markers, including circulating tumor DNA (ctDNA) carrying somatic mutations in genes such as KRAS, EGFR, and TP53 as well as circulating microRNAs whose dysregulation correlates with tumor subtype and stage, have rapidly gained clinical importance. Cellular and vesicular markers such as circulating tumor cells (CTCs) and tumor-derived exosomes preserve molecular signatures of the parent tumor and provide complementary functional information, while emerging metabolic markers such as D-2-hydroxyglutarate (D-2-HG) in IDH-mutant gliomas extend the analyte space further. Clinically, these markers underpin four overlapping functions: early detection, differential diagnosis and subtyping, prognosis and risk stratification, and longitudinal treatment-response monitoring. Their analytical interrogation is nevertheless demanding, since circulating concentrations frequently span six or more orders of magnitude, target species are masked by a vast excess of background molecules, and reliable quantification must be achieved in complex biofluids. It is precisely these analytical demands that biosensor technologies are designed to meet [4,5].

To address these limitations, liquid biopsy has emerged as a minimally invasive alternative that shifts the diagnostic paradigm from static tissue analysis toward dynamic molecular surveillance via accessible body fluids such as blood [6,7]. By analyzing circulating tumor-derived components such as CTCs, ctDNA, and exosomes, liquid biopsy provides a systemic and near real-time window into tumor biology and shows considerable promise for early detection, prognosis, and treatment monitoring [8,9,10]. The notion of dynamic molecular surveillance, however, presupposes a sampling cadence short enough to resolve clinically meaningful biological change. Current evidence on ctDNA-guided treatment-response monitoring supports sampling at two-to-four-week intervals during active therapy and every one to three months during post-treatment surveillance, while minimal residual disease (MRD) monitoring after curative-intent resection typically employs monthly to quarterly sampling. To our knowledge, none of the biosensor platforms reviewed here has yet achieved truly continuous in vivo monitoring of tumor-derived analytes in patients. The practical contribution of biosensor technology is therefore to lower the cost, sample volume, and turnaround time of repeated measurement so that the frequency of conventional liquid biopsy can be raised toward this surveillance ideal. Concrete steps in this direction include the smartphone-connected electrochemical exosome detector, which quantifies CD63-positive exosomes from 5 µL of serum within roughly two hours, and paper-based microfluidic devices that support repeat measurement in community-clinic or at-home settings [11,12].

Biosensor technology has risen to meet this challenge, with electrochemical and optical sensors, particularly fluorescent platforms, at the forefront of clinical translation, while emerging modalities such as SERS, photoelectrochemical (PEC), field-effect transistor (FET), microfluidic, and CRISPR-Cas-based sensors are advancing rapidly [13]. Our own work on fluorescence-based multiplex aptamer sensors [14], biomimetic AIE biosensors [15], and receptor-based fluorescence polarization assays [16,17]. has directly informed the critical perspective of this review.

We focus this review on electrochemical and fluorescent biosensors as the two most clinically mature modalities, and devote a dedicated section to the remaining emerging platforms together with a unified critical comparison across all of them at the end of the manuscript. It is worth noting that the clinical maturity of these two modalities is substantiated by regulatory approvals and commercial adoption. For electrochemical sensors, electrochemiluminescence immunoassay (ECLIA)-based platforms, which represent a specific implementation of electrochemical sensing, have been deployed in clinical laboratories worldwide for routine serum tumor marker testing (e.g., AFP, CEA, PSA). The corresponding detection kits have received regulatory clearances including NMPA Class III certification (China) and IVDR CE marking; as exemplified by one manufacturer receiving IVDR CE certification for 53 electrochemiluminescence-based assay kits including tumor marker tests. Beyond conventional lab-based platforms, a number of emerging electrochemical sensor platforms are approaching commercialization. The Check4 platform (IdentifySensors Biologics), which employs screen-printed graphene electrodes for ctDNA detection, has demonstrated analytical sensitivity comparable to FDA-cleared PCR-based tests (50 copies/mL) and is aiming to offer the assay as a CLIA laboratory-developed test for lung cancer within approximately eight months. On the fluorescence front, commercial adoption is similarly advanced. The flow-fluorescence-based tumor marker detection platform from Tellgen has been deployed in over 1000 hospitals across China, offering a panel of 20 tumor marker assays. Meanwhile, the HIVEN^®^ fluorescence-guided surgical device (Marginum) has completed clinical testing at multiple university hospitals (Oslo, Tampere, Kuopio) and received EU MDR certification (CE mark) as a Class IIb medical device in September 2025. These commercialized and near-commercialized examples collectively support the classification of electrochemical and fluorescent sensors as the two most clinically mature modalities among those reviewed. It should be noted, however, that truly continuous wearable sensor devices for tumor marker monitoring have not yet progressed beyond small-scale feasibility studies to prospective interventional clinical trials.

The next leap in tumor diagnostics is unlikely to come from incremental improvements to single-analyte assays, but rather from strategically leveraging the complementary strengths of these sensing modalities. Electrochemical platforms, prized for their quantitative precision, operational simplicity, and innate compatibility with miniaturization for point-of-care devices, excel at the rapid quantification of protein and nucleic acid biomarkers [18]. In contrast, fluorescent sensors offer unparalleled multiplexing capability through spectral encoding and, uniquely, enable spatially resolved, real-time imaging of molecular processes within living cells, such as telomerase activity or miRNA fluctuations—a capability critical for understanding tumor dynamics at the cellular level [19].

Recent advances in nanoscale engineering are further erasing previous performance boundaries. The emergence of novel materials, such as ultra-small metal nanoclusters (<2 nm), exemplifies this trend. These materials exhibit size-tunable photoluminescence and electrochemiluminescence with exceptional brightness and stability, serving as versatile signal transducers that can bridge and enhance both electrochemical and optical sensing paradigms [20]. Simultaneously, the field is moving beyond the detection of singular, established biomarkers. Research is expanding the liquid biopsy landscape to include emerging targets like circulating cancer-associated fibroblasts, which reflect tumor microenvironment activity, and is increasingly focused on multiplexed profiling of biomarker panels (e.g., combined exosomal surface proteins) to improve diagnostic accuracy and overcome tumor heterogeneity [21].

This review aims to provide a critical synthesis of this evolving landscape. We will first delineate and contrast the technical principles and performance envelopes of major sensor types. We will then delve into the latest innovative strategies in electrochemical and fluorescent biosensing, structured not merely by target biomarker but by the core signal transduction and amplification mechanisms employed (e.g., nucleic acid nanomachines, CRISPR-Cas integration, ratiometric sensing). A key focus will be on how modern designs address specific clinical challenges, such as multiplexing and point-of-care compatibility. Finally, we will discuss the persistent translational hurdles—including standardization, clinical validation, and integration with data analytics—and outline future trajectories toward integrated, multi-modal diagnostic systems that can deliver comprehensive, actionable insights for precision oncology.

## 2. Overview of Sensor Technologies for Cancer Diagnosis

### 2.1. Technical Principles of Different Sensor Types for Tumor Biomarker Monitoring

**Figure 1:**
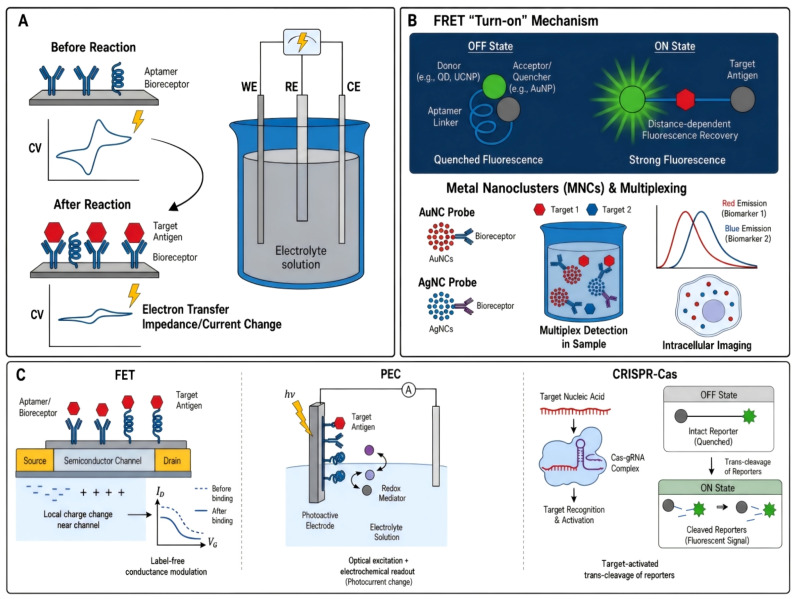
Schematic principles of electrochemical and fluorescent biosensing strategies. (**A**) Electrochemical sensors detect target-induced electron transfer changes (impedance or current) at the electrode interface. (**B**) Fluorescent sensors utilize FRET mechanisms for signal recovery and metal nanoclusters for multiplexed biomarker detection. (**C**) Additional transduction modalities, including FET, PEC, and CRISPR-Cas systems, operate through label-free conductance modulation, light-induced photocurrent readout, and target-activated reporter cleavage.

The biosensor platforms covered in this review fall into seven principle-level categories according to their signal-transduction physics (Figure 1). Electrochemical sensors transduce binding events into current or potential changes at a modified electrode, while fluorescent sensors translate the same recognition events into changes in emission intensity, wavelength, lifetime, or polarization. Surface-enhanced Raman scattering (SERS) sensors rely on plasmonic hot spots to enhance the molecular-fingerprint Raman signal of surface-attached species, whereas photoelectrochemical (PEC) sensors spatially decouple optical excitation from electrical readout to suppress background interference [22]. Field-effect transistor (FET) biosensors operate in a label-free manner by modulating the conductance of a semiconductor channel upon target binding. Microfluidic chip-based sensors integrate fluid handling with any of the above readouts on a single device, providing automation and multiplexing within microliter sample volumes. CRISPR-Cas-based sensors couple the trans-cleavage activity of Cas12a or Cas13a to fluorescent, electrochemical, or colorimetric reporters, enabling sequence-specific detection with single-mismatch discrimination. The following subsections describe the operating principles of each modality in turn.

#### 2.1.1. Electrochemical Biosensors

Electrochemical biosensors utilize biological recognition elements such as enzymes, antibodies, and nucleic acid aptamers as probes, which are immobilized on electrode surfaces. An electrochemical workstation converts the specific binding events between the recognition elements and the target analytes into measurable changes in current or voltage signals [20,23]. The fundamental principle is that when the target (e.g., CTCs or ctDNA) binds to the recognition elements on the electrode surface, it alters the interfacial electron transfer properties, thereby generating a responsive current or potential shift. This technology offers advantages including simple instrumentation, high sensitivity, and low cost, making it particularly suitable for the rapid detection of trace tumor biomarkers in body fluid samples [24,25]. In recent years, the performance of electrochemical sensors has been significantly enhanced through the introduction of nanomaterials (e.g., gold nanoparticles, graphene, MXenes) and nucleic acid signal amplification strategies (e.g., rolling circle amplification, hybridization chain reaction, DNA walker) [26,27].

#### 2.1.2. Fluorescent Biosensors

Fluorescent biosensors employ fluorescence signals as their output. Mechanisms such as fluorescence resonance energy transfer (FRET) and metal-enhanced fluorescence convert molecular recognition events into changes in fluorescence wavelength or intensity [28,29]. These sensors typically consist of a fluorescence donor (e.g., quantum dots, upconversion nanoparticles, organic dyes) and an acceptor (e.g., quenchers, gold nanoparticles). Recently, metal nanoclusters have garnered significant attention as a novel class of fluorescent nanomaterials. Comprising only a few to several tens of metal atoms, they offer advantages such as small size, good biocompatibility, facile synthesis, and tunable fluorescence properties (from the visible to near-infrared regions) via surface ligand modification [19]. The presence of the target analyte alters the distance or energy transfer efficiency between the donor and acceptor, leading to a “turn-on” or “turn-off” fluorescence signal. By functionalizing metal nanoclusters with different emission wavelengths (e.g., Ag nanoclusters, Au nanoclusters) with distinct recognition molecules (e.g., aptamers), multiplexed fluorescent sensors capable of simultaneously responding to multiple tumor biomarkers can be constructed [30]. Fluorescent sensors offer high sensitivity and the capability for real-time, in situ imaging. They are not only applicable for detecting biomarkers in body fluids but can also be integrated with nanocarriers (e.g., MnO2 nanosheets, DNA tetrahedrons) to enable in situ monitoring of intracellular telomerase activity or miRNA expression levels in living cells [31]. However, a major challenge remains the efficient delivery of exogenous probes into live cells while maintaining cell viability [32].

#### 2.1.3. Surface-Enhanced Raman Scattering (SERS) Sensors

SERS exploits the localized surface plasmon resonance of noble-metal nanostructures to amplify the Raman scattering of molecules adsorbed on the metal surface, with reported enhancement factors ranging from 10^6^ to 10^9^. The resulting spectrum acts as a molecular fingerprint and supports both label-free identification and reporter-tagged multiplexed quantification. By engineering substrates that concentrate the electromagnetic field at well-defined “hotspots” and by selecting Raman reporters with distinguishable spectral signatures, several tumor markers can be detected simultaneously in a single measurement [33].

#### 2.1.4. Photoelectrochemical (PEC) Sensors

PEC sensors couple optical excitation to electrochemical readout. Photosensitive materials such as semiconductor heterojunctions, quantum dots, and nanocomposites are deposited on a working electrode and convert incident light into a measurable photocurrent that depends on the binding state of surface-immobilized recognition probes [34]. Because the excitation source and the detection signal are of different physical natures, background interference is suppressed and femto- to sub-femtogram sensitivity becomes accessible for protein, nucleic-acid, and exosome targets.

#### 2.1.5. Field-Effect Transistor (FET) Biosensors

In an FET biosensor, the recognition element is immobilized on the gate or channel surface of a semiconductor device, and the binding of a charged target perturbs the local electrostatic potential and thereby the channel conductance [35]. The readout is intrinsically label-free and rapid, and the device geometry is compact and amenable to wafer-scale fabrication, which makes FET platforms attractive for miniaturized multiplexed assays.

#### 2.1.6. Microfluidic Chip-Based Sensors

Microfluidic biosensors integrate fluid manipulation at the microliter scale with one or more transduction modalities, including fluorescence, electrochemistry, and colorimetry, on a single chip. The platform supports automated sample preparation, multiplexed analysis, and high-throughput operation while consuming minimal sample and reagent volumes. When combined with portable optical or electrochemical readers, microfluidic chips can be deployed in point-of-care and resource-limited settings.

#### 2.1.7. CRISPR-Cas-Based Sensors

CRISPR-Cas-based sensors translate sequence-specific recognition by a guide-RNA-programmed Cas effector, most commonly Cas12a or Cas13a, into a measurable signal through the trans-cleavage of reporter probes. Once the target is recognized, the activated Cas enzyme indiscriminately cleaves nearby fluorescent, electrochemical, or colorimetric reporters and produces an amplified output with single-mismatch discrimination [36]. The approach is particularly well suited to circulating nucleic-acid markers such as ctDNA and microRNAs.

## 3. Development and Application of Electrochemical Biosensors for Tumor Biomarker Detection

Electrochemical biosensors convert biorecognition events into measurable electrical signals. They have made significant progress in tumor liquid biopsy because of their high sensitivity, rapid response, easy miniaturization, and low cost. This chapter systematically reviews the current research and application status of electrochemical detection strategies, focusing on three key biomarkers: CTCs, ctDNA, and exosomes.

### 3.1. Electrochemical Biosensors for Circulating Tumor Cell Detection

#### 3.1.1. Capture Interfaces Based on Antibodies and Aptamers

Early electrochemical CTC sensors primarily relied on antibody recognition. The epithelial cell adhesion molecule (EpCAM), overexpressed on the surface of most epithelial-derived CTCs, is a commonly used capture target. For example, a label-free electrochemical immunosensor based on polyamidoamine dendrimers for detecting EpCAM on the surface of HepG2 hepatocellular carcinoma cells, achieving a detection limit of 2.1 × 10^3^ cells/mL [37]. However, issues related to the stability and cost of antibodies have driven researchers to seek alternative probes. Nucleic acid aptamers, as chemically synthesized single-stranded DNA or RNA molecules, offer advantages such as ease of synthesis, good stability, and flexible modification. A sensor based on an EpCAM aptamer was constructed, which utilizing rolling circle amplification and hemin/G-quadruplex DNAzyme for dual-signal amplification, significantly lowering the detection limit to 1 cell/mL [38].

CTCs are cells shed from primary or metastatic tumors into the circulatory system, serving as direct evidence of tumor metastasis. However, their extremely low abundance in peripheral blood (only a few cells per milliliter) poses significant challenges to the sensitivity and specificity of detection technologies. Electrochemical sensors have achieved ultrasensitive CTC detection by combining efficient cell capture strategies with signal amplification techniques.

#### 3.1.2. Multi-Target Collaborative Recognition and Signal Amplification Strategies

To improve the specificity of CTC capture and reduce non-specific adsorption from background cells like leukocytes, multi-target collaborative recognition strategies have proven effective. a dual-recognition electrochemical sensor simultaneously targeting both EpCAM (using an antibody) and mucin 1 (using an aptamer) were designed [37]. This strategy requires target cells to co-express both biomarkers for effective capture, thereby enhancing recognition specificity while achieving ultra-high sensitivity comparable to single-target strategies (1 cell/mL). These studies collectively demonstrate that through the optimization of recognition elements and the integration of efficient signal amplification techniques, electrochemical sensors have become a powerful tool for CTC detection.

### 3.2. Electrochemical Biosensors for ctDNA Detection

CtDNA consists of fragmented DNA released into the bloodstream upon apoptosis or necrosis of tumor cells, carrying tumor-specific mutation information. Its extremely low concentration, masked by a vast background of wild-type DNA, makes ultra-high sensitivity and specificity the core requirements for ctDNA detection.

#### 3.2.1. Sensing Mechanisms Based on DNA Self-Assembly and Isothermal Amplification

Traditional ctDNA detection methods such as digital PCR or next-generation sequencing are often time-consuming, costly, and require complex instrumentation. Electrochemical biosensors offer a new pathway for rapid, low-cost detection [39]. Among them, signal amplification strategies based on DNA self-assembly and isothermal amplification play a key role. Li et al. designed a target ctDNA-triggered “DNA walker” system [10]. Based on rolling circle amplification, this system initiates the continuous movement of the “walker” on the electrode surface upon target recognition, releasing signal molecules and achieving a detection limit of 0.29 fM. A ratiometric electrochemical sensor was developed [40], which utilizes target ctDNA to cyclically activate DNAzyme, causing a change in the signal ratio of two electrochemical reporters (methylene blue and ferrocene). This effectively counters interference from environmental fluctuations, pushing the detection limit to an extremely low level of 25 aM.

#### 3.2.2. High-Specificity Detection Empowered by the CRISPR/Cas System

The high specificity of the CRISPR/Cas system has been introduced into electrochemical sensing platforms. A CRISPR/Cas9 triggered entropy driven strand displacement reaction system was constructed, which immobilized on a three-dimensional graphene/gold-platinum-palladium nanoflower composite [41]. The specific cleavage of the target sequence by the Cas9/sgRNA complex triggers a cascade amplification reaction, enabling not only ultra-sensitive detection of ctDNA but also multiplexed mutation analysis, with a recovery rate of 91.75–111.50% in serum samples, demonstrating significant potential for clinical application.

### 3.3. Electrochemical Biosensors for Exosome Detection

Exosomes are nanoscale extracellular vesicles secreted by cells, carrying rich molecular information (proteins, nucleic acids, etc.) from their parent cells, making them promising non-invasive diagnostic biomarkers. The main challenges lie in their efficient isolation from complex biofluids and highly sensitive quantification of their surface-specific protein markers.

#### 3.3.1. Strategies Based on Aptamers and DNA Signal Amplification

Electrochemical sensing commonly uses the exosomal transmembrane protein CD63 as a universal capture target. For instance, an electrochemical aptasensor for the detection of gastric cancer derived exosomes was constructed [42]. Anti-CD63 antibodies immobilized on the electrode surface capture exosomes, followed by a gastric cancer exosome-specific aptamer triggering a rolling circle amplification reaction. This produces numerous G-quadruplex structures that bind hemin to form DNAzymes, which catalyze H_2_O_2_ to generate an electrical signal, achieving a detection limit of 9.54 × 10^7^ particles/mL. For cancers with high heterogeneity such as breast cancer, detecting a single marker is often insufficient. A magnetically mediated multiplexed protein analysis sensor was developed. The sensor capable of simultaneously detecting four proteins (CD63, MUC1, HER2, and EpCAM) on the surface of breast cancer-derived exosomes, enabling the detection of 1.0 × 10^7^ particles/μL in patient serum, thereby providing richer information for cancer subtyping [43].

#### 3.3.2. Novel Functional Materials and Portable Devices

The application of novel functional materials has greatly enhanced sensor performance. Such as, a non-metallic porphyrin-based metal–organic framework (MOF) through a simple Zn^2+^ post-modification strategy could significantly enhanced the electrochemiluminescence intensity. It construct an ultra-sensitive exosome sensor with a detection limit of 9.08 × 10^3^ particles/μL, without requiring additional signal amplification steps [44]. To meet POCT demands, portable devices are also rapidly developing. A miniature electrochemical detector connected to a smartphone was developed, capable of quantifying CD63-positive exosomes in 5 μL serum samples within 2 h, with a detection limit of 7.23 ng, providing a feasible solution for immediate diagnosis [11]. Notably, electrochemical microfluidic paper-based analytical devices (µPADs) offer an extremely promising platform for the low-cost, on-site detection of exosomes. One study reported a paper-based device fabricated by laser cutting and carbon ink electrodes, employing a sandwich immunoassay for exosome detection with a limit of detection of 9.3 × 10^7^ particles/mL [12]. This platform integrates sample transport, capture, and electrochemical detection on a single paper chip, simplifying operational procedures and reducing costs.

### 3.4. Electrochemical Biosensors for Conventional Protein Tumor Biomarker Detection

Electrochemical biosensors are highly mature for detecting conventional protein tumor biomarkers (e.g., AFP, CEA, PSA, CA125, HER2) in serum and other body fluids. The introduction of various nanomaterials, particularly dendrimers, has opened new dimensions for performance enhancement. These highly branched nanostructures can serve as ideal signal amplification carriers or electrode modification materials due to their abundant surface functional groups [45].

#### 3.4.1. Signal Amplification and Integrated Platforms Based on Dendrimers

Dendrimers, especially PAMAM, can covalently load numerous enzyme molecules (e.g., horseradish peroxidase, HRP), electron mediators (e.g., ferrocene), or metal nanoparticles onto their surfaces, thereby transforming a single biorecognition event into a powerful electrochemical signal. For example, PAMAM dendrimers were utilized to co load CdTe@CdS quantum dots and recognition antibodies, constructing an electrochemiluminescence immunosensor for the detection of CA125. This strategy achieved high sensitivity and a wide linear range (0.1–400 U/mL) [38]. Such a “one carrier, multiple signal agents” approach greatly enhances detection sensitivity. Similarly, for the breast cancer biomarker CA15-3, sensors employing PAMAM dendrimers conjugated with ruthenium complexes have achieved an impressively low detection limit [46].

#### 3.4.2. Integration with Paper-Based Microfluidic Platforms

Electrochemical microfluidic paper-based analytical devices (µPADs) represent a revolutionary paradigm for the on-site and low-cost detection of traditional tumor biomarkers, effectively addressing the critical need for portability and operational simplicity in resource-limited settings. The inherent capillarity of paper drives fluid flow without external pumps, enabling a self-contained “sample-in, answer-out” operation. In a typical design, a sample droplet applied to an inlet zone wicks through a hydrophilic channel patterned by hydrophobic barriers. As the sample passes over a working electrode pre-modified with capture probes (e.g., antibodies or aptamers), target analytes are specifically immobilized, while unbound matrix components continue to flow towards an absorbent pad. This intrinsic filtration mechanism elegantly eliminates the need for the tedious and error-prone washing steps characteristic of conventional assays like ELISA [12].

The versatility of electrochemical µPADs is reflected in their compatibility with diverse detection strategies. Label-free approaches, which often monitor changes in charge-transfer resistance via electrochemical impedance spectroscopy [4] using redox mediators, offer simplicity and directness. Conversely, labeled strategies employing enzyme conjugates (e.g., horseradish peroxidase, alkaline phosphatase) or electroactive nanomaterials (e.g., methylene blue, ferrocene) linked to detection antibodies achieve exceptional sensitivity through catalytic or intrinsic redox signal amplification, typically measured via differential pulse voltammetry or square wave voltammetry.

A significant advantage of µPADs is their inherent suitability for multiplexed analysis. By spatially patterning multiple detection zones or utilizing distinguishable electroactive labels, simultaneous quantification of several biomarkers from a single sample becomes feasible. For instance, a μPAD for the label free, concurrent detection of carcinoembryonic antigen (CEA) and neuron-specific enolase (NSE) was developed [47], demonstrating the platform’s capacity for comprehensive profiling.

Importantly, the analytical performance of these ostensibly simple paper devices can rival that of benchtop instruments, largely due to strategic nanomaterial integration. Modification of paper-based electrodes with conductive nanomaterials such as gold nanoparticles (AuNPs), reduced graphene oxide (rGO), or carbon nanotubes dramatically enhances electron transfer kinetics, electrode surface area, and bioreceptor loading capacity. This is exemplified by a paper-based aptasensor for prostate-specific antigen (PSA), where an AuNPs/rGO/thionine nanocomposite modification yielded a remarkable detection limit of 10 pg/mL across a broad linear range (0.05–200 ng/mL) [8]. Similarly, an origami-based µPAD configured for epidermal growth factor receptor (EGFR) detection achieved a sensitivity of 5 pg/mL, underscoring the potential of these platforms for ultra-sensitive analysis with minimal sample volume [48].

#### 3.4.3. Summary of Performance for Protein-Marker Electrochemical Platforms

Table 1 summarizes representative electrochemical sensors for conventional protein tumor biomarkers, with particular emphasis on dendrimer-based amplification and paper-microfluidic integration. A cross-modality critical comparison that places these electrochemical platforms alongside fluorescent, SERS, PEC, FET, microfluidic, and CRISPR-Cas-based sensors is presented in Section 6. Electrochemical sensors face three underappreciated challenges. Electrode fouling by proteins and cells in whole blood alters effective surface area and charge transfer resistance; anti-fouling coatings only partially mitigate this. Signal drift from mediator leaching and surface morphology changes limits suitability for continuous monitoring. Reproducibility across sensor batches, especially for nanomaterial-modified electrodes (graphene, MXenes, AuNPs), typically shows 10–20% CV, necessitating batch-specific calibration. Most reported sensors are validated in buffer or diluted serum; data on undiluted whole blood performance are scarce. Standardized fabrication protocols and routine reporting of electrode-to-electrode CV for a standard redox probe would greatly enhance clinical translatability.

## 4. Development and Application of Fluorescent Biosensors for Tumor Marker Detection

Fluorescent biosensors detect variations in fluorescence signals to monitor tumor markers. These sensors provide several significant advantages, including high sensitivity, excellent selectivity, rapid response times, and superior performance in optical imaging and spatial applications resolution. Their function relies on the specific binding of target molecules, such as proteins, nucleic acids, or exosomes, to recognition elements like antibodies or aptamers. This binding modifies measurable fluorescence properties, including intensity, wavelength, and polarization, enabling both qualitative and quantitative analysis of targets [49]. In recent years, research has shifted from optimizing single-analyte sensitivity to developing intelligent sensing systems. These systems are designed for multiplexed detection, perform reliably in complex biological settings, and help bridge in vitro diagnostics and in vivo imaging.

Early studies using organic dyes and conventional quantum dots have produced valuable results, but these materials are limited by photostability and biocompatibility [50,51]. Recent advances in fluorescent nanomaterials present promising alternatives. Among these, near-infrared II probes confer significant advantages. Their emission occurs within the tissue’s “optical transparency window”, substantially diminishing light scattering and absorption while reducing autofluorescence interference. This facilitates high-resolution imaging of deep tissues in vivo [52,53]. Another significant category is aggregation-induced emission materials. These materials exhibit weak fluorescence when dispersed; however, they emit strongly upon aggregation. This non-light characteristic enables the development of highly sensitive detection systems with minimal background [54]. Other nanomaterials, including carbon dots, perovskite quantum dots, and upconversion nanoparticles, also demonstrate significant potential. Their tunable optical properties, low toxicity, or anti-Stokes emission make them suitable for building high-performance multimodal sensing platforms [55,56,57]. To improve measurement reliability in complex samples, internal calibration strategies are often used. Ratiometric fluorescence detection and fluorescence polarization assist in mitigating systematic errors induced by changes in environmental factors [58,59]. The design strategies, targeting mechanisms, and imaging performance of these advanced probe materials are summarized in Table 2.

Advances in sensor technology continue to broaden the applications of fluorescent biosensors. In vitro diagnostics increasingly use these sensors, which are increasingly integrated with microfluidic and paper-based chip platforms. This integration enables automated, high-throughput, and multiplexed detection of tumor markers [60,61]. The development of smartphone-integrated sensing systems is particularly noteworthy. It enables highly sensitive tumor marker testing to move beyond the laboratory. These systems are now applicable to community healthcare and personal health management [62]. Fluorescent biosensors are also capable of targeting specific and dynamic biomarkers. For instance, they can detect molecules such as PD-L1 on the surfaces of tumor cells or analyze constituents of the tumor microenvironment. This capability enables real-time visual monitoring of processes such as tumor immune status and metabolic activity [63]. Looking forward, a promising direction is the development of “theranostic” probes. These agents combine high-sensitivity diagnostic capabilities with precise optical therapeutic capabilities. This integration enables simultaneous diagnosis and treatment [64]. A critical comparison reveals inherent trade-offs. FRET offers high spatial resolution but requires precise fluorophore positioning and suffers from spectral overlap and photobleaching. Fluorescence polarization enables wash-free assays but is limited to small analytes (<100 kDa) and turbid media. Aggregation-induced emission (AIE) materials provide low-background “turn-on” sensing yet demand controlled aggregation kinetics. Upconversion nanoparticles (UCNPs) eliminate autofluorescence for in vivo imaging but have low quantum yields (<1%) and potential toxicity. Among nanomaterials, quantum dots (QDs) offer high quantum yield and photostability but raise toxicity concerns; cadmium-free QDs are safer but less bright. Metal nanoclusters are biocompatible and ultra-small but exhibit modest quantum yields (1–10%) and oxygen sensitivity. AIEgens circumvent concentration quenching but introduce aggregation control complexity. Carbon dots are low-cost and nontoxic but suffer from poorly understood emission mechanisms and batch variability. No single nanomaterial is universally optimal; choice must be guided by target analyte and application setting, with standardized reporting of quantum yield, photostability, and batch reproducibility urgently needed.

Three analytical challenges critically limit the practical utility of fluorescent biosensors. Stability: Organic dyes photobleach within minutes; QDs and UCNPs are photostable but prone to colloidal aggregation in biofluids; metal NCs oxidize in solution within days. Most sensors are validated in buffer, but performance in serum often degrades rapidly. Matrix interference: Serum proteins (60–80 mg/mL) cause non-specific adsorption and protein corona formation, altering recognition properties [65,66,67]. Autofluorescent species (NADH, flavins) and inner-filter effects produce false signals. Ratiometric and time-resolved designs partially mitigate these issues but cannot fully eliminate matrix effects without extensive sample pretreatment. Reproducibility: Batch-to-batch variation in nanoparticle synthesis is rarely quantified; reported coefficients of variation (CVs) in spiked serum (typically 5–15%) are often from optimal conditions. Inter-laboratory validation is virtually absent. We recommend future studies report storage stability (signal retention over weeks), matrix effect quantification (serum vs. buffer), and batch-to-batch CV from ≥3 independently synthesized batches. Current research extends beyond single-protein markers to include the capture and analysis of whole functional units. These units include circulating tumor cells, exosomes, and extracellular vesicles [68,69]. Intelligent fluorescent biosensors can detect multiple parameters simultaneously. They measure factors such as pH, reactive oxygen species, and enzyme activity within the tumor microenvironment [70]. This multiparameter approach enables multidimensional analysis of tumor heterogeneity and progression [71,72]. The field still faces challenges, including long term biosafety, in vivo stability, and clinical translation. However, continuous innovation in materials, sensing mechanisms, and design concepts drives progress. In vitro diagnostics continue to advance through integration, with sensors increasingly combined with microfluidic and paper based platforms for automated, high throughput, and multiplexed detection of tumor markers.

**Table 2:** Performance parameters of fluorescent biosensors for tumor marker detection.

Tumor Markers	Sensing Technique/Method	Identification Component	Probe/Material	Linear Range	Detection Limit	Sample	Ref.
PSA	Specific FL sensing/Fluorescence quenching	PSA aptamers	ZGO:Mn NRs	10 fg/mL–1 ng/mL	8.9 fg/mL	human serum	[73]
CA15-3	Specific FL sensing/Fluorescence quenching	CA15-3 monoclonal antibody	CdTe@MPA	0.09–0.91 U/mL	0.027 U/mL	human serum	[56]
CEA	Specific FL sensing/sense amplifier	CEA aptamers	OPC	0.1–2.5 ng/mL	0.1 ng/mL	saliva	[74]
HER2	Specific FL sensing/Fluorescence quenching	HER2 aptamers	ZIF-8@COU	0.05–10 ng/mL	0.0005 ng/mL	human serum	[75]
AFP	Specific FL sensing/sandwich structure	AFP aptamers	3VPD	0.1–1000 ng/mL	0.05 ng/mL	human serum	[60]
CA199	Specific FL sensing/sandwich structure	CA199 aptamers	3VPD	0.1–1000 U/mL	0.09 U/mL	human serum	[60]
CEA	label-free fluorescence/sense amplifier	CEA aptamers	Apt@AuNP	0.1–2.5 nM	0.03 nM	solution	[76]
D-2-HG	Specific FL sensing/sense amplifier	D2HGlo	FRET	1–50 μM	308 nM	cells	[77]
GPC3	Specific FL sensing/Fluorescence resonance energy transfer	(Fe)3O4/GO–GPC3	GPC3/AuCDs-GPC3Apt	5–100 ng/mL	3.01 ng/mL	human serum	[55]
TPE-TAs	Specific FL sensing/Fluorescence quenching	TPE–TAs aptamers	GO	0.68–30.4 pM	0.57 pm	exosomes	[78]
CA-125	Specific FL sensing/sandwich structure	CA–125 aptamers	NaYF4:Yb/Tm and Ag	5–100 ng/mL	120 pg/mL	solution	[57]
VEGF	Specific FL sensing/Fluorescence polarization	DNA aptamers	FAM/G-quadruplex aptamer-VEGF	0.32–5.0 nM	0.32 nM	human serum	[58]
CEA	Label-free fluorescence/sense amplifier	CEA aptamers	p-acid@SiO	0.1–100 ng/mL	0.04 ng/mL	human serum	[79]
PSA	Specific FL sensing/Fluorescence resonance energy transfer	PSA aptamers	PMMA OPC	0.1–10 ng/mL	0.01 ng/mL	solution	[80]

Abbreviations: PSA: Prostate-specific-antigen; CA15-3: Cancer antigen 15-3; CEA: Carcinoembryonic antigen; HER2: Human epidermal growth factor receptor 2; AFP: Alpha-fetoprotein; CA199: Carbohydrate antigen199; GPC3: Glypican-3; TPE-TAs: Tetraphenylethylene fluorophores; CA-125: Cancer antigen 125; VEGF: Vascular endothelial growth factor; D-2-HG: D-2-hydroxyglutarate; OPC: Opal photonic crystal; 3VPD: 3D vertical-flow paper-based device; FRET: Fluorescence resonance energy transfer; GO: Graphene oxide; PMMA OPC: Polymethyl methacrylate-based opal photonic crystal.

## 5. Development and Application of Other Tumor Marker Detection Sensors

**Figure 2:**
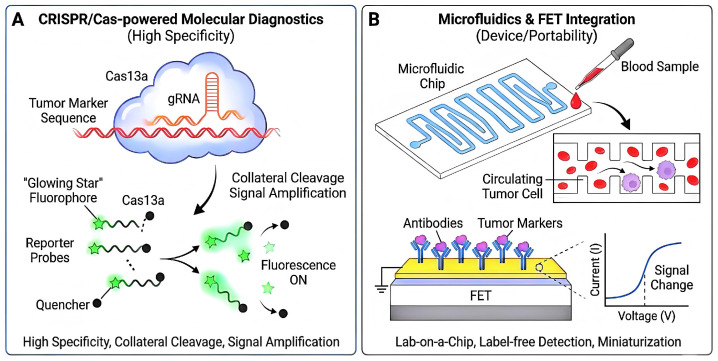
Advanced enabling technologies for enhanced sensing performance. (**A**) CRISPR/Cas systems leverage collateral cleavage activity to achieve high-specificity signal amplification. (**B**) Integrated platforms combining microfluidics and Field-Effect Transistors (FET) enable precise sample handling and label-free, miniaturized detection.

In addition to electrochemical and fluorescent biosensors, other sensing technologies are used for tumor marker detection (Figure 2). These methods offer useful approaches with their own strengths. They exhibit high specificity, can be readily integrated into devices, and enable simultaneous detection of multiple markers. Together, they form a diverse and advanced set of analytical tools. Their main features are summarized in Table 3.

### 5.1. Surface-Enhanced Raman Scattering (SERS) Sensors

Building on the SERS principle introduced in Section 2.1.3, application-oriented research has concentrated on substrate engineering and reporter design for tumor-marker quantification, in which the molecular fingerprint provided by the enhanced Raman spectrum supports highly specific identification [94]. The simultaneous detection of multiple markers has been achieved by combining hotspot-rich plasmonic substrates with spectrally distinguishable Raman reporter molecules, which together enable multiplexed quantification within a single measurement [82,95,96]. Two practical challenges nevertheless continue to limit clinical translation. The reproducible large-scale fabrication of SERS substrates with consistent enhancement performance remains technically demanding, and the development of reliable quantitative protocols for use in complex biological matrices such as serum is still an active area of methodological research [97].

### 5.2. Microfluidic Chip-Based Sensors

Beyond the integration principle outlined in Section 2.1.6, microfluidic biosensors have found particular utility in early cancer diagnostics, liquid-biopsy workflows, and rapid diagnostic testing, where their ability to handle minute sample volumes with automated multiplexed readout offers clear advantages over benchtop assays [98]. The compact format also lends itself to integration with smartphones and other portable devices, opening applications in home-based care and community health programs [99]. When equipped with optical or electrochemical detectors, microfluidic chips are especially effective at profiling multiple biomarkers from limited samples and at isolating rare targets such as circulating tumor cells and exosomes, which together establish them as a key technology for next-generation point-of-care diagnostics [91,100,101]. The principal barriers to wider adoption remain the complexity of chip design and the cost of scalable manufacturing [102].

### 5.3. Photoelectrochemical Sensors

Following the operating principle described in Section 2.1.4, where the spatial separation of optical excitation and electrical readout intrinsically suppresses background and enhances sensitivity [103], PEC sensors have advanced rapidly through the engineering of photoactive electrode materials such as semiconductor heterojunctions, quantum dots, and nanocomposites, all of which improve photo-to-current conversion efficiency and amplify the analytical response [104,105]. In practical performance, PEC platforms have detected established protein markers including carcinoembryonic antigen and prostate-specific antigen at femtogram and even sub-femtogram levels, well below the sensitivity ceiling of most conventional immunoassays [106]. Their adaptability extends well beyond protein markers, with successful demonstrations on ctDNA, tumor-associated microRNAs, exosomes, and CTCs, which substantially broadens the analyte scope of liquid biopsy [107,108,109]. Several challenges nevertheless remain before clinical deployment. Photo-to-electrical conversion efficiency is not always optimal, nonspecific adsorption from complex matrices such as serum can compromise specificity, and environmental factors such as dissolved oxygen concentration influence both signal stability and quantitative accuracy [110].

### 5.4. Field-Effect Transistor (FET) Biosensors

Building on the operating principle outlined in Section 2.1.5, FET biosensors directly transduce target binding into a measurable change in semiconductor channel conductance, and their label-free, real-time, and miniaturizable readout makes them well suited to tumor-marker detection [111]. Recent FET research has emphasized recognition-element miniaturization and multiplexing for cancer diagnostics. The use of compact recognition probes such as antigen-binding fragments has enhanced effective signal transduction within the Debye length, enabling detection of alpha-fetoprotein in serum at concentrations as low as 100 pg/mL with high sensitivity and specificity [88]. The intrinsic device geometry of FET arrays also lends itself to multiplexed analysis, and the simultaneous detection of CYFRA 21-1 and neuron-specific enolase has been demonstrated for lung-cancer subtyping [89]. Several technical hurdles continue to constrain clinical translation, including signal attenuation by Debye screening in physiologically relevant ionic media, nonspecific adsorption of background molecules, and long-term device stability. Continued progress in surface chemistry and system-level integration will be essential before FET biosensors can support reliable multiplexed detection in routine clinical use.

### 5.5. CRISPR-Cas-Based Sensors

Building on the operating principle of CRISPR-Cas-based sensors described in Section 2.1.7, application-driven research has concentrated on highly sensitive detection of tumor-associated nucleic acids such as circulating tumor DNA, microRNAs, and messenger RNAs, with the recognition output coupled to fluorescent, electrochemical, or colorimetric reporters [112,113]. Here we critically assess four dimensions: enzyme choice, isothermal amplification integration, specificity limitations, and clinical validation.

Cas12a vs. Cas13a. Cas12a (Class 2 Type V) targets DNA, exhibits low-turnover trans-nuclease activity, and is preferred for ctDNA and mutation detection. Cas13a (Class 2 Type VI) targets RNA, shows high-turnover trans-RNase activity, and is ideal for miRNA and viral RNA. Both can be activated by non-canonical triggers, but their distinct kinetics guide sensor design: Cas12a for DNA liquid-biopsy analytes, Cas13a for direct RNA detection without reverse transcription [114].

Integration with isothermal amplification. Pre amplification (using RPA or LAMP) is often needed to convert trace biomarkers into detectable concentrations. One-pot RPA-CRISPR/Cas12a assays achieve LODs as low as 3.43 aM (miRNA-21) and 1.0 × 10^2^ EVs/μL (exosomes). However, amplification-free CRISPR diagnostics face challenges from slow Cas kinetics and diffusion limits; solutions include Cas engineering, novel signal transduction, and digital spatial confinement. Critical integration challenges include non-specific amplification, multiplexing standardization, and robust performance in resource-limited settings.

Specificity limitations. Off-target cleavage—arising from guide RNA hybridization to imperfectly matched sequences—is a recognized limitation, particularly pronounced in eukaryotic backgrounds where SNPs can compromise on-target activity. Additionally, Cas12a requires a PAM sequence (e.g., 5′-TTN-3′) adjacent to the target, restricting target choice. Engineered mitigation strategies include AND-logic gated systems (0.1% VAF sensitivity), PNA-mediated Cas13a (PRICE, ~10 fM LOD), RECO-Cas (0.01% VAF), and NRAS PASEA (0.01% mutant allele fraction) [115,116,117].

Real clinical applications and persistent gaps. The CRISPR/Cas12a-G4 biosensor for breast cancer ctDNA was validated on 48 clinical samples (100% specificity, 92% sensitivity, 4 aM LOD). A dual RPA-CRISPR/Cas13a platform for colorectal cancer achieved 84% sensitivity and 85% specificity. RECO-Cas detected KRAS, EGFR, and PIK3CA mutations in plasma with 90.48% sensitivity and 100% specificity. Notably, the first registered interventional trial (ChiCTR2600118972) is evaluating a CRISPR/Cas12a-based 3D DNA nanomachine for lung cancer ctDNA. Despite these advances, a 2026 systematic review critically notes that most CRISPR-Cas cancer diagnostic studies remain limited to spiked buffer or serum, lacking prospective multi-center validation against hard endpoints. Matrix interference, lack of standardized protocols, and limited multiplexing remain major barriers. As discussed in Section 6.6 and Section 6.8, ultra-low LODs under idealized conditions should not be equated with clinical readiness.

## 6. Critical Comparison Across Sensor Modalities

Having surveyed electrochemical, fluorescent, SERS, photoelectrochemical, field-effect transistor, microfluidic, and CRISPR-Cas-based platforms in the preceding sections, we now place these modalities side by side along the performance axes most relevant to clinical translation (Table 4). The discussion below focuses primarily on electrochemical and fluorescent sensors, which together account for the majority of reported tumor-marker assays, and integrates the remaining modalities where they provide distinctive advantages. Key parameters considered include sensitivity and limit of detection, specificity and matrix robustness, detection speed and amenability to point-of-care testing, applicable scenarios, and clinical relevance beyond the binary detection of biomarker presence.

### 6.1. Sensitivity and Limit of Detection (LOD)

Sensitivity is a core parameter for evaluating sensor performance. Overall, electrochemical sensors, employing various signal amplification strategies (e.g., nucleic acid amplification, nanomaterial catalysis), have achieved detection limits ranging from attomolar (aM, 10–18 M) to single-cell levels. For instance, a ratiometric electrochemical sensor developed, based on DNAzyme cycling amplification, achieved an LOD of 25 aM for ctDNA [40]. A dual recognition electrochemical sensor was designed, which implemented a dual-recognition strategy using EpCAM antibody and mucin 1 (MUC1) aptamer, achieving an LOD as low as 1 cell/mL for CTCs [37]. Fluorescent sensors also exhibit excellent sensitivity, showing particular advantages in ctDNA and intracellular biomarker detection. For example, AgInS2/ZnS quantum dots coupled with hybridization chain reaction amplification fluorescent sensor achieved an LOD of 53 aM for ctDNA [118]. A homogeneous detection strategy based on CdTe quantum dots was reporte, with a fluorescent LOD of 0.25 cells/mL [119].

### 6.2. Specificity and Anti-Interference Capability

The specificity of electrochemical sensors often relies on the high-affinity interactions between antibody–antigen or aptamer-target pairs [25]. However, non-specific adsorption from complex matrices in body fluids can cause signal interference. Recent advances, such as employing dual-recognition probes (e.g., antibody-aptamer combinations) or incorporating the specific cleavage function of the CRISPR/Cas system, have significantly improved the selectivity of electrochemical sensors [120]. The specificity of fluorescent sensors depends heavily on the precision of probe design and the energy transfer efficiency of the FRET pair. For instance, fluorescent probes based on DNA tetrahedrons or molecularly imprinted polymers can effectively exclude non-target interferences through steric hindrance or template recognition, enhancing detection reliability in complex samples like serum [119].

### 6.3. Detection Speed, Portability, and Prospects for Point-of-Care Testing (POCT)

Electrochemical sensors demonstrate significant advantages in the field of POCT due to their rapid response and great potential for device miniaturization. For example, a paper-based electrochemical sensor and a smartphone-connected electrochemical detection platform, both enabled rapid and portable quantitative analysis of exosomes [121]. Although fluorescent sensors offer comparable detection speeds, their reliance on complex optical readout equipment has historically limited their portability. However, with the development of smartphone-integrated fluorescence detection modules and visual readout strategies (e.g., colorimetric-fluorescence dual-mode output based on CdTe quantum dots), significant progress has been made in the development of fluorescent POCT devices [122].

### 6.4. Applicable Scenarios and Core Advantages

As discussed in Section 3 and Section 6.1, the core advantages of electrochemical sensors lie in their simple instrumentation, rapid response, and ease of miniaturization, making them highly suitable for development into portable POCT devices. Research has successfully integrated electrochemical sensors with paper-based platforms or smartphones to achieve on-site, rapid analysis of biomarkers like exosomes [121]. In contrast, the outstanding advantages of fluorescent sensors are their superior spatial resolution and real-time imaging capabilities. Beyond detecting biomarkers in body fluids, fluorescent sensors can be combined with functionalized nanocarriers (e.g., MnO2 nanosheets, DNA tetrahedrons) to achieve in situ, real-time, and dynamic monitoring of intracellular processes like telomerase activity or miRNA expression—a capability that is challenging to realize with electrochemical methods [119].

### 6.5. Clinical Relevance Beyond Detection: Subtype Discrimination, Risk Stratification, and Prognosis

Reliable detection of biomarker presence is necessary but not sufficient for clinical impact, and we therefore evaluate the reviewed platforms against three higher-order clinical functions. Cancer subtype discrimination is increasingly accessible to multiplexed sensors that quantify several markers from a single sample. The magnetic-bead multiplex aptasensor, for example, simultaneously quantifies CD63, MUC1, HER2, and EpCAM on breast-cancer-derived exosomes and yields a four-dimensional signature capable of separating HER2-positive from triple-negative subtypes. The dual-channel FET sensor for CYFRA 21-1 and NSE likewise supports differentiation between squamous-cell and small-cell lung carcinoma in principle. Beyond protein-marker quantification, mutation profiling extends the clinical utility of sensors to therapy stratification: the CRISPR/Cas9-coupled electrochemical ctDNA sensor performs multiplexed somatic-mutation analysis with high specificity and addresses the clinical need for genotype-guided therapy selection such as EGFR-TKI eligibility and KRAS-mutation exclusion. Quantitative biosensor outputs become genuinely prognostic when their dynamic range encompasses clinically validated cutoffs. CellSearch-defined CTC counts at or above five per 7.5 mL of blood are associated with poorer overall survival in metastatic breast, prostate, and colorectal cancer, and several aptamer- and antibody-based electrochemical platforms reviewed here achieve limits of detection of one cell per milliliter or below, well within the actionable range. For ctDNA, longitudinal change rather than single-timepoint absolute level is the most robust prognostic indicator, which further reinforces the importance of low-cost, rapid, and repeatable assays. Despite this progress, most of the reviewed platforms remain validated only at the analytical-performance level on small spiked-serum or banked-cohort studies, and prospective longitudinal validation against hard clinical endpoints such as recurrence, progression-free survival, and overall survival is still scarce. Closing this gap through multi-center clinical-utility studies with pre-specified decision thresholds is the most pressing next step before subtype-classification and prognostic claims can be formally substantiated.

### 6.6. Quantitative Benchmarking and Clinical Relevance of Detection Limits

A direct quantitative meta-analysis across the reviewed studies is precluded by substantial heterogeneity in experimental conditions. Key variables include sample matrix (buffer, diluted serum, undiluted plasma, whole blood), sample volume (microliters to milliliters), signal amplification strategy (enzymatic, nanomaterial-catalyzed, nucleic acid amplification), and LOD definition (signal-to-noise ratio of 3, 3σ, or other criteria). Consequently, the LOD values reported in Table 1, Table 2 and Table 3 should be interpreted as context-dependent figures of merit rather than absolute rankings.

To assess clinical relevance, we compare reported LODs against clinically actionable thresholds for representative tumor markers. For serum protein markers (AFP, CEA, PSA, CA125), clinical cutoffs typically lie in the ng/mL to μg/mL range (e.g., PSA cutoff 4 ng/mL for biopsy referral; AFP cutoff 20 ng/mL for hepatocellular carcinoma surveillance). Most electrochemical and fluorescent sensors achieve LODs in the pg/mL or even fg/mL range, substantially below these thresholds, indicating that sensitivity is rarely the limiting factor for such markers. The greater challenge is maintaining specificity and reproducibility at near-threshold concentrations. For ctDNA bearing specific mutations (e.g., EGFR T790M, KRAS G12D), clinically relevant variant allele frequencies (VAFs) depend on the clinical context: early-stage disease and minimal residual disease (MRD) monitoring demand detection down to 0.01–0.1% VAF, whereas tissue-based genotyping for late-stage disease typically relies on a 1% reporting threshold. Several electrochemical and CRISPR-Cas-based sensors report LODs in the aM range (10^−18^ M), which is mathematically equivalent to approximately 0.6 copies/μL. Assuming a typical total cfDNA concentration of 10 ng/μL of plasma (approx. 3000 genome-equivalents per μL), an aM-level LOD corresponds to a theoretical VAF of approximately 0.1–1% depending on the exact cfDNA yield and the definition used, a range that meets MRD detection requirements. However, these LODs are typically determined in buffer or synthetic oligonucleotide-spiked samples; performance at low VAFs in the presence of a vast excess (10^3^–10^4^-fold) of wild-type DNA—a realistic clinical scenario—is seldom reported. For CTCs, the clinical benchmark is well-established: patients with metastatic breast cancer with ≥5 CTCs per 7.5 mL blood have significantly worse prognosis, and most reviewed electrochemical sensors achieve detection limits of 1 cell/mL or better, which is clinically adequate. For exosomes, however, a universally accepted quantitative clinical threshold does not yet exist; the clinical utility often relies on qualitative detection or relative abundance rather than absolute quantitation against a fixed cutoff. Indeed, the first FDA-approved exosome-based liquid biopsy test (ExoDx Prostate) exemplifies this approach by reporting a risk score rather than an exosome count threshold.

### 6.7. Translational Barriers: Regulatory Approval, Standardization, and Scalability

While the analytical performance of the reviewed biosensors has advanced considerably, their translational path remains obstructed by regulatory, standardization, scalability, and economic barriers that are often underemphasized in the literature. This subsection critically examines these hurdles.

Regulatory pathways and classification. In the U.S., most novel tumor marker biosensors—particularly those with untested clinical claims or novel transduction mechanisms—would initially fall under Class III (requiring Premarket Approval, PMA), unless a substantial equivalence (510(k)) predicate device exists or a De Novo classification is granted. However, a recent FDA proposal (November 2025) seeks to reclassify specific nucleic acid-based oncology companion diagnostics from Class III to Class II with special controls, potentially easing the pathway for certain ctDNA and NGS-based tests. In the EU, the In Vitro Diagnostic Regulation (IVDR, EU 2017/746) classifies most tumor marker tests intended for treatment decisions as Class C (moderate-to-high risk). This requires conformity assessment by a Notified Body, extensive clinical evidence, and post-market surveillance. Notably, liquid biopsy tests for cancer detection that support treatment decisions are specifically designated as Class C under IVDR classification rules. The transition from the former IVDD to IVDR has dramatically increased the regulatory burden: approximately 80% of IVD products now require Notified Body involvement (compared to 15% previously), with average certification timelines for Class C devices ranging from 18 to 24 months and costs reaching tens to hundreds of thousands of euros.

Standardization and interlaboratory reproducibility. Clinical adoption further demands adherence to established performance standards such as those from the Clinical and Laboratory Standards Institute (CLSI). CLSI EP05-A3 defines precision parameters including repeatability and reproducibility across instruments, operators, and time, while CLSI EP17 and MM06 provide guidance on limit-of-detection determination. However, the reviewed literature rarely reports analytical validation against these standards, and interlaboratory reproducibility studies—a prerequisite for regulatory submission—remain virtually absent. Studies have shown that even with semiconductor manufacturing technology to improve batch consistency, achieving CLSI-compliant reproducibility requires systematic optimization of fabrication parameters, bioreceptor immobilization, and stringent quality controls.

Manufacturing scalability and yield. Scalable production of nanomaterial-modified electrodes—the backbone of many high-performance electrochemical sensors—remains a formidable challenge. While screen-printing is an economical and practical alternative for mass production, industrial-scale manufacturing data reveal pressing limitations: one detailed analysis of a commercial screen-printed carbon electrode production line reported an overall process yield of only 41.5%, with primary defects including unprinted tracks, line width deviations exceeding specifications, and paste accumulation during printing. These defect rates translate directly into higher per-unit costs and reduced commercial viability. Emerging roll-to-roll and advanced electrode printing strategies aim to address these challenges but require further optimization and validation.

Technology readiness levels (TRL). Applying the standardized TRL scale (1 = basic principles, 9 = operational system) reveals a significant gap between research prototypes and clinical products. The majority of the electrochemical, fluorescent, and SERS-based platforms reviewed in this paper operate at TRL 3–4 (experimental proof-of-concept or laboratory validation under simulated conditions). Portable aptasensor prototypes and nanomaterial-based sensors typically reach TRL 4–5 when tested with spiked clinical matrices but generally lack formal regulatory approval and independent multi-site validation. A recent survey of aptamer-based electrochemical biosensors for biomarker detection explicitly notes that none had obtained FDA, CE, or SWGDRUG approval at the time of reporting, with all studies restricted to single-laboratory validation. Advancing beyond TRL 6 (system prototype demonstration in relevant environment) will require large-cohort clinical validation, rigorous quality management systems (ISO 13485), and formal regulatory review.

Translational costs and economic viability. Even when technical performance meets clinical requirements, the path to commercialization is economically daunting. Regulatory submission alone—including analytical validation studies, clinical performance studies, technical documentation, and quality system implementation—can cost several hundred thousand to over one million euros for Class C devices under IVDR, with total development costs including design, manufacturing scale-up, and multi-center trials often exceeding several million euros. For point-of-care devices intended for near-patient use, additional validation for operator variability, environmental robustness, and storage stability further increases costs. Economic analyses suggest that for liquid biopsy-based diagnostics to achieve widespread adoption, cost per test must be substantially reduced compared to centralized laboratory assays, particularly for applications requiring frequent monitoring. Ultimately, cost-effectiveness of novel biosensors will depend not only on manufacturing costs but also on long-term clinical outcomes and healthcare resource utilization—data that are largely absent in the sensor development literature.

In summary, while the reviewed sensors demonstrate remarkable analytical performance, their clinical translation is constrained by a convergence of regulatory, standardization, scalability, and economic barriers. Overcoming these will require not only technological innovation but also proactive regulatory engagement, adherence to international standards, and rigorous health economic modeling from the earliest stages of development.

### 6.8. From Buffer to Bedside: A Reality Check on Analytical Performance

A critical observation across the reviewed literature is that the majority of reported detection limits (often in the aM/fM range) are determined in buffer or synthetic samples, under idealized conditions. While these figures demonstrate technological sensitivity, they do not accurately predict performance in clinical settings. To provide a realistic assessment, we distinguish three levels of validation:

Buffer-based performance: Most studies report LODs in PBS or Tris buffer. These values are typically 10–100× lower than those obtained in complex matrices due to the absence of fouling, autofluorescence, or enzymatic degradation. Such data serve as proof-of-concept but lack clinical relevance.

Spiked serum/plasma performance: A smaller subset of studies validates their sensors in pooled human serum or plasma spiked with known concentrations of the target. Here, LODs often increase by 1–2 orders of magnitude, and recovery rates may deviate from 100% due to matrix effects (protein corona, pH shifts, nucleases). These results are more informative but still do not reflect patient heterogeneity.

Real clinical validation: Only a handful of studies (e.g., those involving patient-derived samples with gold-standard comparison) have moved beyond spiked matrices. In such cases, specificity, positive predictive value, and inter-patient variability become critical. Notably, most sensors reviewed have not been tested against clinically relevant thresholds in authentic patient cohorts, and none have undergone prospective multi-center validation. We therefore caution readers against equating ultra-low LODs in buffer with clinical readiness. Future work must explicitly report performance at each level and prioritize validation in biobanked or prospectively collected patient specimens.

**Table 4:** Comparative summary of sensor modalities for tumor marker detection: advantages, limitations, technology readiness level (TRL), cost, POCT potential, and clinical validation status.

Modality	Key Advantages	Main Limitations	TRL (1–9)	Cost per Test (Reagent/Device)	POCT Potential	Clinical Validation Status
Electrochemical	High sensitivity (aM–fM), simple instrumentation, miniaturizable, quantitative, low power	Electrode fouling, signal drift, batch-to-batch variability (CV 10–20%), limited multiplexing	4–6	Low ($1–10 for disposable sensors; high for ECLIA analyzers)	High (smartphone-connected, paper-based)	ECLIA platforms routinely used; emerging POC sensors lack multi-center validation
Fluorescence (dyes/QDs)	High sensitivity, multiplexing via spectral encoding, real-time imaging capability	Photobleaching, autofluorescence interference, toxicity (Cd-based QDs), complex optics	4–5	Moderate ($10–50 for kits; high for imagers)	Moderate (smartphone adapters exist, but optics bulky)	Flow-fluorescence platform commercial (Tellgen); intraoperative device (HIVEN^®^) CE-marked
Fluorescence (UCNPs/AIEgens)	No autofluorescence (UCNPs), high signal/background (AIE), deep-tissue imaging	Low QY (UCNPs < 5%), aggregation control needed (AIE), synthesis complexity	3–4	High (UCNP synthesis)	Low (requires NIR laser)	Preclinical only; no clinical test
SERS	Molecular fingerprint specificity, multiplexing, high sensitivity (fM–aM)	Poor reproducibility across batches (CV 10–25%), expensive substrates, complex instrumentation	3–4	High (noble-metal substrates, Raman spectrometer)	Low (instrument size, cost)	No clinical test; some LDT prototypes for gastric cancer (research)
PEC	Ultra-low background, high sensitivity (fg/mL), decoupled excitation/detection	Environmental interference (O_2_, pH), complex electrode modification, slow response	3–4	Moderate (photoactive materials)	Low (requires light source and potentiostat)	No clinical validation
FET	Label-free, real-time, wafer-scale fabrication, small footprint	Debye screening, non-specific binding, device-to-device variation (CV 12–30%)	4–5	Moderate (semiconductor fabrication)	Moderate (compact, but requires stable gate bias)	No clinical test for tumor markers; some for other diseases
Microfluidic chip	Low sample/reagent volume, automated multiplexing, integrates multiple detection modes	Complex design, expensive prototyping, scaling challenges, low yield	4–5	High to low (depends on complexity)	Moderate (if integrated with smartphone readout)	Some LDTs for CTC enumeration (CellSearch-like); no POC chip approved for tumor markers
CRISPR-Cas	High specificity (single-base discrimination), isothermal amplification, programmable	Off-target effects, PAM dependence, Cas enzyme stability, multiplexing difficult	3–4	Low (Cas proteins, guide RNAs)	High (paper-based, lateral flow)	One registered clinical trial (ChiCTR2600118972); no approved product

TRL definitions: 1–2 basic research; 3–4 proof-of-concept, lab validation; 5–6 prototype in relevant environment; 7–8 system complete, clinical validation; 9 commercial operational. POCT Potential: Low (lab-only), Moderate (portable but requires some infrastructure), High (handheld, minimal steps).

## 7. Conclusions and Future Perspectives

Research in medicine has shown that biomarkers such as DNA, RNA, proteins, and cells are intrinsically linked to the initiation, progression, and metastasis of tumors. Sensors engineered to detect these biomarkers offer high sensitivity, real-time responsiveness, and multiplexed detection, making them highly promising for clinical deployment. In recent years, sensors based on electrochemical, fluorescent, SERS, photoelectrochemical, field-effect transistor, microfluidic, and CRISPR-Cas-based technologies have each contributed to earlier tumor diagnosis and improved monitoring of disease progression. Ongoing advances in nanomaterials, molecular probes, and microfabrication techniques further enhance sensor performance, with a focus on higher sensitivity, multi-target analysis, and increased portability.

Looking ahead, tumor marker sensors are evolving toward multimodal integration, intelligent operation, and improved clinical utility. The integration of optical, electrical, magnetic, and acoustic sensing modalities can establish unified platforms that acquire complementary data, thereby enhancing the reliability of diagnostic outcomes. The incorporation of artificial intelligence for real-time data analysis will further enable adaptive, smart sensing systems that respond to dynamic changes in the physiological microenvironment. Advances in biocompatible materials and micro-nanofabrication are also enabling the development of implantable and wearable sensor devices. Through close interdisciplinary collaboration and a focus on unmet clinical needs, the next generation of tumor marker sensors is expected to combine diagnosis, continuous monitoring, and treatment guidance into practical, clinically ready systems.

**Figure 3:**
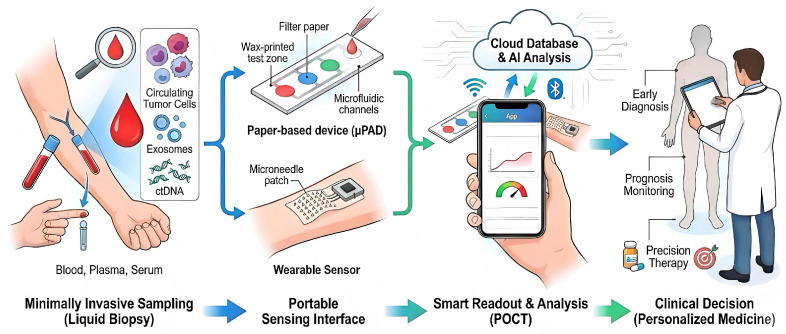
Future clinical workflow from liquid biopsy to intelligent point-of-care testing (POCT). Minimally invasive sampling of circulating biomarkers is integrated with portable sensing devices (e.g., µPADs, wearables) and AI-assisted smartphone analysis to facilitate real-time precision oncology.

While the foregoing perspectives highlight promising directions, a more critical examination of several under-addressed themes is warranted. Commercialization barriers extend beyond regulatory approval. They also include manufacturing scalability, high translation costs, and the scarcity of prospective multi-center validation. Digital interoperability—the ability of different sensor platforms to exchange and interpret data seamlessly—remains largely ignored. Most prototypes operate as standalone devices with proprietary data formats, impeding integration into electronic health records or multi-modal diagnostic workflows. Integration with artificial intelligence (AI) holds promise for adaptive sensing, pattern recognition, and real-time decision support, yet most studies report only basic signal processing. Deep learning models for multiplexed sensor data remain in early research stages, and their generalizability across different patient populations and sensor batches is unproven. Finally, standardization of biomedical data—including pre-analytical variables (sample collection, storage, processing), meta-data annotations, and quality control metrics—is conspicuously absent, hindering both inter-laboratory reproducibility and the development of large-scale training datasets for AI. Addressing these themes will require interdisciplinary collaboration among sensor engineers, data scientists, regulatory specialists, and clinical laboratorians, moving beyond proof-of-concept demonstrations toward system-level integration and validation in real-world health systems (Figure 3).

## Figures and Tables

**Table 1:** Summary of electrochemical biosensors for the detection of tumor biomarkers.

Tumor Marker(s)	Sensing Method	Identification Component	Signal Material	Linear Range	LOD	Sample	Ref.
CTCs	CV/EIS	EpCAM antibody	Polyamidoamine dendrimer	N/S	2.1 × 10^3^ cells/mL	HepG2 cells	[37]
	SWV	EpCAM aptamer	Hemin/G-quadruplex DNAzyme (via RCA)	5–10^7^ cells/mL	1 cell/mL	Spiked blood	[38]
	DPV	Dual-recognition (Anti-EpCAM + Anti-MUC1 aptamer)	Branched PtAuRh trimetallic nanospheres	5–1 × 10^6^ cells/mL	1 cell/mL	Patient blood	[37]
ctDNA	Amperometry	DNA walker probe	Enzymatic amplification (via RCA)	N/S	0.29 fM	Synthetic oligo	[10]
	Ratiometric DPV	DNAzyme activation probe	Methylene blue/Ferrocene (cycling)	N/S	25 aM	Synthetic oligo	[40]
	EIS/DPV	CRISPR/Cas9-sgRNA complex	Entropy-driven strand displacement	N/S	N/S	Human serum	[41]
Exosomes	DPV	Anti-CD63 Ab + Gastric cancer aptamer	Hemin/G-quadruplex (via RCA)	N/S	9.54 × 10^7^ particles/mL	Cell culture supernatant	[42]
	Amperometry	Multiplex Abs (CD63, MUC1, HER2, EpCAM)	Magnetic bead-host-guest recognition	N/S	1.0 × 10^7^ particles/μL	Patient serum	[43]
	ECL	Anti-CD63 Ab	Zn^2+^-modified porphyrin MOF	N/S	9.08 × 10^3^ particles/μL	Cell culture supernatant	[44]
	Amperometry (POCT)	Anti-CD63 Ab	HRP catalysis (smartphone readout)	N/S	7.23 ng (in 5 μL)	Human serum	[11]
	DPV (µPAD)	Anti-CD63 Ab	Carbon ink electrode	10^8^–10^10^ particles/mL	9.3 × 10^7^ particles/mL	Human plasma/serum	[12]
AFP	SWV	Anti-AFP antibody	Prussian blue-CNTs@polydopamine	0.005–80.000 ng/L	0.001 ng/L	Human serum	[8]
	CV	Anti-AFP antibody (Sandwich)	Pd octahedral and APTES-M-CeO-GS composite	N/S	0.033 ng/L	Human serum	[8]
CEA	SWV	Anti-CEA antibody (Sandwich)	Cu-MOFs/polydopamine nanocarrier	0.002–200.000 μg/L	0.003 μg/L	Human serum	[8]
	DPV (Label-free µPAD)	Anti-CEA aptamer	NG-Thionine-AuNPs nanocomposite	0.01–500 ng/mL	2 pg/mL	Human serum	[47]
PSA	DPV (Label-free µPAD)	Anti-PSA aptamer	AuNPs/rGO/Thionine nanocomposite	0.05–200 ng/mL	10 pg/mL	Human serum	[8]
HER2	DPV	Anti-HER2 aptamer	Reduced graphene oxide-chitosan	0.5–75.0 μg/L	0.21 μg/L	Human serum	[8]
CA125	ECL	Anti-CA125 antibody	PAMAM-CdTe@CdS-Ab_2_ composite	0.1–400 U/mL	0.1 U/mL	Human serum	[46]
CA15-3	ECL	Anti-CA15-3 antibody	PAMAM-Ru(bpy)_3_^2+^ composite	10^−4^–10^2^ U/mL	10^−7^ U/mL	Human serum	[46]
EGFR	DPV (Label-free µPAD)	Anti-EGFR aptamer	Thionine redox indicator	0.05–200 ng/mL	5 pg/mL	Human serum	[48]
SCCA	DPV	Anti-SCCA antibody	PtFe alloy/N-doped carbon nanoflowers	0.010–10.000 μg/L	0.003 ng/L	Human serum	[8]
OPN	CV/EIS (Label-free)	Anti-OPN antibody	AuNPs modified electrode	0.001–1000.000 ng/L	0.005 μg/L	Spiked human serum	[8]

Abbreviations: PSA: Prostate specific antigen; CA15-3: Cancer antigen 15-3; CEA: Carcinoembryonic antigen; HER2: Human epidermal growth factor receptor 2; AFP: Alpha-fetoprotein; CA-125: Cancer antigen 125; GO: Graphene oxide.

**Table 3:** Performance parameters of other types of tumor marker detection sensors.

Sensing Technique/Method	Tumor Markers	Identification Component	Probe/Material	Linear Range	Detection Limit	Sample	Ref.
Surface enhanced raman spectroscopy	VEGF	VEGF aptamers	SEHGNs	0.1–1000 ng/mL	1–10 pg/mL	solution	[81]
Surface enhanced raman spectroscopy	PDGF-B	PDGF-Baptamers	Au/SiNUA	10^–14^–10^–9^ M	2.18 fM	human serum	[82]
Surface enhanced raman spectroscopy	Thrombin	Thrombin aptamers	Au/SiNUA	10^–14^–10^–9^ M	2.79 fM	human serum	[82]
AlN thin film bulk acoustic resonators	MUC1	MUC1 aptamers	Streptavidin	30–500 nM		solution	[83]
ZnO film bulk acoustic resonators	MUC1	MUC1 aptamers	Streptavidin	20–500 nM	20 nM	solution	[84]
Bio-functionalized plasmonic metasurfaces	CEA	CEA aptamers	Gold nanobumps	10–87 ng/mL	10 ng/mL	human serum	[85]
Visible light photoelectrochemical sensor	CEA	CEA aptamers	AuNP/PDDA-G-SnO_2_	0.005–10 ng/mL	0.036 pg/mL	human serum	[86]
Ratiometric electrochemical immunity Biosensors	CEA	CEA aptamers	Polythionine-gold (PTh-Au)/K_3_[Fe(CN)_6_]	0.005–40 ng/mL	2.2 pg/mL	human serum	[87]
Field effect transistor Biosensors	AFP	AFP aptamers	ΔVg	100 pg/mL–1 µg/mL	100 pg/mL	solution	[88]
Field effect transistor Biosensors	AFP	AFP aptamers	ΔVg	1 ng/mL–1 µg/mL	1 ng/mL	human serum	[88]
Field effect transistor Biosensors	CYFRA 21-1	CYFRA 21-1 antibody	ΔVg	1 ng/mL–1 μg/mL	1 ng/mL	human serum	[89]
Field effect transistor Biosensors	NSE	NSE antibody	ΔVg	1ng/mL–1 μg/mL	10 ng/mL	human serum	[89]
Microfluidic chip Biosensors	SARS-CoV-2 N	SARS-CoV-2N antigen	μPADs	0.008–0.266 mg/mL	8 μg/mL	solution	[90]
Microfluidic chip Biosensors	DAS	DAS aptamers	OECT-MIPs	1 nM–10 μM	0.8 nM	solution	[91]
CRISPR/Cas12a based electrochemical biosensor	Target DNA	Target DNA	E-CRISPR		0.68 aM	DNA	[92]
CRISPR/Cas12a based electrochemical biosensor	Target DNA	Target DNA	E-CRISPR		26 cfu/mL	flammulina velutipes	[92]
CRISPR/Cas13a triggered-DNA walker amplified SERS sensor	miRNA-106a	miRNA-106a	Zn2+-SERS-DNA	100 aM–1 nM	53.16 aM	human serum	[93]

Abbreviations: VEGF: Vascular endothelial growth factor; PDGF-B: Platelet-derived growth factor subunit B; MUC1: Human mucin-1; CEA: Carcinoembryonic antigen; AFP: Alpha-fetoprotein; CYFRA 21-1: Cytokeratin 19 fragment antigen21-1; NSE: Neuron specific enolase; SARS-CoV-2 N: Severe acute respiratory syndrome coronavirus 2 nucleocapsid; DAS: N12-diacetylspermine; SEHGNs: Silica-encapsulated hollow gold nanospheres; Au/SiNUA: Gold-coated silicon nanowire array; AuNP/PDDA-G-SnO_2_: Gold nanoparticles/poly(diallyldimethylammonium chloride)-graphene-SnO_2_ composite; μPADs: Microfluidic paper-based analytical devices; OECT-MIPs: Organic electrochemical transistor—molecularly imprinted polymers; E-CRISPR: Electrochemical CRISPR.

## Data Availability

This is a review article. No primary research results, software or code have been included and no new data were generated or analyzed as part of this review.

## Abbreviations

The following abbreviations are used in this manuscript:

AFP	Alpha-fetoprotein
aM	attomolar
AuNPs	Gold Nanoparticles
CA125	Cancer Antigen 125
CA15-3	Cancer Antigen 15-3
CA199	Carbohydrate Antigen 199
Cas	CRISPR-associated
CEA	Carcinoembryonic Antigen
CNTs	Carbon Nanotubes
CRISPR	Clustered Regularly Interspaced Short Palindromic Repeats
ctDNA	circulating Tumor DNA
CTCs	Circulating Tumor Cells
CV	Cyclic Voltammetry
CYFRA 21-1	Cytokeratin 19 Fragment Antigen 21-1
DAS	N12-diacetylspermine
D-2-HG	D-2-hydroxyglutarate
DPV	Differential Pulse Voltammetry
ECL	Electrochemiluminescence
EGFR	Epidermal Growth Factor Receptor
ELISA	Enzyme-Linked Immunosorbent Assay
EIS	Electrochemical Impedance Spectroscopy
FET	Field-Effect Transistor
FRET	Fluorescence Resonance Energy Transfer
GPC3	Glypican-3
GO	Graphene Oxide
HRP	Horseradish Peroxidase
HepG2	Human Hepatocellular Carcinoma Cell Line
HER2	Human Epidermal Growth Factor Receptor 2
LOD	Limit of Detection
MUC1	Human Mucin 1
MOF	Metal–Organic Framework
MIPs	Molecularly Imprinted Polymers
miRNA	microRNA
MXenes	Transition Metal Carbides, Nitrides and Carbonitrides
NSE	Neuron-Specific Enolase
N-CDs	Nitrogen-Doped Carbon Dots
Ni NCs	Nickel Nanoclusters
OPN	Osteopontin
OPC	Opal Photonic Crystal
OECT	Organic Electrochemical Transistor
PDGF-B	Platelet-Derived Growth Factor Subunit B
PD-L1	Programmed Death-Ligand 1
PAMAM	Polyamidoamine
PEC	Photoelectrochemical
PCR	Polymerase Chain Reaction
PDDA	Poly(diallyldimethylammonium chloride)
POCT	Point-of-Care Testing
PMMA	Polymethyl Methacrylate
PSA	Prostate Specific Antigen
RCA	Rolling Circle Amplification
rGO	reduced Graphene Oxide
SCCA	Squamous Cell Carcinoma Antigen
SERS	Surface-Enhanced Raman Scattering
SEHGNs	Silica-Encapsulated Hollow Gold Nanospheres
sgRNA	single-guide RNA
SWV	Square Wave Voltammetry
TPE-TAs	Tetraphenylethylene Fluorophores
UCNP	Upconversion Nanoparticle
VEGF	Vascular Endothelial Growth Factor
µPADs	Microfluidic Paper-Based Analytical Devices
3VPD	3D Vertical-Flow Paper-Based Device
QD	Quantum Dot
